# Extreme fluctuations in ambient salinity select for bacteria with a hybrid “salt-in”/”salt-out” osmoregulation strategy

**DOI:** 10.3389/frmbi.2023.1329925

**Published:** 2024-01-08

**Authors:** Danny Ionescu, Luca Zoccarato, Pedro J. Cabello-Yeves, Yaron Tikochinski

**Affiliations:** ^1^ Plankton and Microbial Ecology, Leibniz Institute of Freshwater Ecology and Inland Fisheries (IGB), Stechlin, Germany; ^2^ Institute of Computational Biology, University of Natural Resources and Life Sciences 24 (BOKU), Vienna, Austria; ^3^ Core Facility Bioinformatics, University of Natural Resources and Life Sciences (BOKU), Vienna, Austria; ^4^ Evolutionary Genomics Group, Departamento de Producción Vegetal y Microbiología, Universidad Miguel Hernández, Alicante, Spain; ^5^ Cavanilles Institute of Biodiversity and Evolutionary Biology, University of Valencia, Valencia, Spain; ^6^ School of Life Sciences, University of Warwick, Coventry, United Kingdom; ^7^ The School of Marine Sciences, The Ruppin Academic Center, Michmoret, Israel

**Keywords:** osmoregulation, Dead Sea, underwater springs, metagenome-assembled genome (MAG), salinity fluctuation

## Abstract

Abundant microbial biofilms inhabit underwater freshwater springs of the Dead Sea. Unlike the harsh (i.e., over 35% total dissolved salts) yet stable environment of the basin, the flow rate of the springs changes with random amplitude and duration, resulting in drastic shifts in salinity, pH, and oxygen concentrations. This requires the organisms to continuously adapt to new environmental conditions. Osmotic regulation is energetically expensive; therefore, the response of the biofilm organisms to rapid and drastic changes in salinity is interesting. For this purpose, we studied the metagenome of an enrichment culture obtained from a green biofilm-covered rock positioned in a spring. We obtained metagenome-assembled genomes (MAGs) of *Prosthecochloris* sp. (*Chlorobiales*), *Flexistipes* sp. (*Deferribacterales*), *Izemoplasma* (*Izemoplasmatales*), *Halomonas* sp. (*Oceanospirillales*), and *Halanaerobium* (*Halanaerobiales*). The MAGs contain genes for both the energetically cheaper “salt-in” and more expensive “salt-out” strategies. We suggest that the dynamic response of these bacteria utilizes both osmoregulation strategies, similar to halophilic archaea. We hypothesize that the frequent, abrupt, and variable-in-intensity shifts in salinity, typical of the Dead Sea spring system, select for microorganisms with scalable adaptation strategies.

## Introduction

Halophilic bacteria and Archaea thrive in a wide array of salinities ranging from seawater to saturated brines. The environments where the sodium chloride (NaCl) concentration is close to saturation and where extreme halophiles thrive are common globally (Atanasova et al., 2013) and harbor relatively low biodiversity. Such environments provide a suitable environment for studying biological, ecological, and evolutionary questions regarding the limits of microbial life on Earth (Ma et al., 2010). Moreover, halophilic bacteria are of special interest for biotechnological industries, which require bioprocessing under high salt concentrations and non-sterile conditions, in an energy- and resource-saving way, for the production of cheaper materials and fuels (Moreno et al., 2016; Chen and Jiang, 2018). Additionally, mechanosensitive channels used for osmoregulation by bacteria are relevant models in the field of mechanobiology, applied to the study of diseases such as cardiopathies or muscular dystrophy (Martinac et al., 2014).

To account for the external osmotic pressure halophile microorganisms have adopted two main strategies. The first, commonly used by hyper-halophiles, was named the “salt-in” strategy as it involves a high intracellular concentration of salts, mainly potassium. The second strategy, “salt-out”, is more common among moderate halophiles, as well as marine microbes, and relies on the accumulation of small organic compounds, generally referred to as compatible solutes, e.g., glycine betaine and trehalose (Kunte et al., 2002; Scanlan et al., 2009; Kunte, 2009).

The type of osmoregulatory mechanism used by an organism has various implications. On the one hand, the salt-in strategy is energetically less expensive than the salt-out alternative, in which compatible solutes need to be synthesized or acquired from the environment if available (Oren, 2008). On the other hand, the salt-in strategy requires the presence of an acidic proteome capable of functioning in the presence of molar concentration of intracellular salts (Deole et al., 2013), whereas the salt-out strategy does not require any special adaptations.

Traditionally it was believed that the type of osmoregulation employed by an organism follows a phylogenetic separation by which extreme halophilic Archaea make use of the salt-in strategy (Becker et al., 2014), while moderate and slight halophilic bacteria and eukaryotes use the salt-out alternative (Roessler and Muller, 2001; Oren, 2005). Nevertheless, an increasing number of halophilic bacteria have been shown to utilize the salt-in strategy, the first of which was *Salinibacter ruber* (Oren, 2013a). Subsequently, two sister proteobacterial taxa, *Halorhodospira halochloris* and *Halorhodospira halophila*, were found to utilize different osmoregulation strategies, one using the salt-out and the other the salt-in strategy, respectively (Oren, 2013b). Interestingly, the suggestion that *H. halochloris* does not have an acidic proteome based on isoelectric gel analysis was later disproved from its genome sequence, which showed that the transition of *H. halochloris* to the salt-out strategy may have happened late in the evolution of this genus (Deole et al., 2013).

The Dead Sea is a hypersaline lake with a concentration of ca. 350 g L^−1^ total dissolved salts consisting mainly of 2 M Mg^2+^, 1.5 M Na^+^, and 0.5 M Ca^2+^ and a matching ca. 7 M Cl^−^ (Oren, 2010). The microbial life in the Dead Sea has been described in a series of papers in the first half of the 20th century covering both prokaryotes and eukaryotes (Wilkansky, 1936; Elazari-Volcani, 1940; Elazari-Volcani, 1943; Elazari-Volcani, 1944). Unlike other aquatic environments, including hypersaline systems, due to the high concentration of divalent cations and possibly due to the low concentrations of available phosphorus, bacterial concentrations in the lake’s water are low, ranging between 10^4^ and 10^5^ cells mL^−1^ under normal conditions (Ionescu et al., 2012). Occasionally, during strong winter seasons the input of freshwater increases, leading to a dilution of the upper water layer, which in turn results in a bloom of the green alga *Dunaliella* sp. A consequence of this is that the number of prokaryotes in the water column increases as well (Kaplan and Friedmann, 1970; Oren, 1985; Oren and Gurevich, 1995).

**Figure 1:**
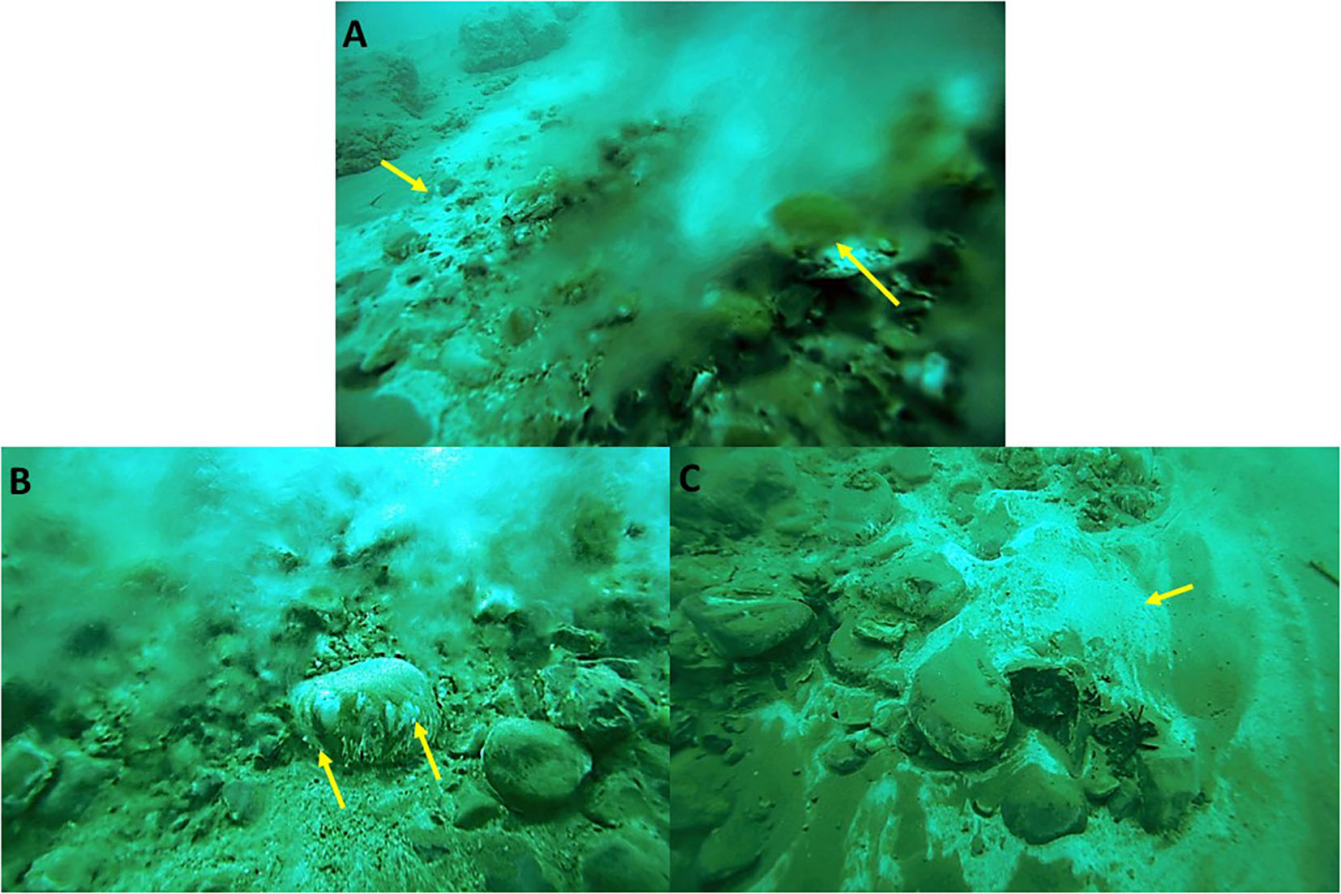
White and green biofilms as they appear in the Dead Sea underwater springs (examples are marked by arrows). Often, white biofilms are found at the peripheries of the main outlet of springs, whereas stones covered in green and white biofilms are found in the main flow **(A)**. A close-up of a stone shows that the biofilms are located on the underside of the stone where they benefit from an average lower salinity (Häusler et al., 2014a) **(B)**. A white biofilm on the sediment photographed at a time where no flow could be observed, demonstrating the vast fluctuations in the flow regime **(C)**.

Submarine groundwater discharge has been documented in the Dead Sea for over a decade (Ionescu et al., 2012; Mallast et al., 2013). Nevertheless, the effects it has on the biota of the Dead Sea near the water outlets was only recently investigated (Ionescu et al., 2012). Extensive biofilms on the sediments and stones at the freshwater/saltwater interface have been discovered (**Figure 1**). Interestingly, the water flow in these underwater springs is characterized by extreme fluctuations that occur at a seemingly random amplitude and frequency (Häusler et al., 2014a, **Figure S1**). The ambient water salinity is directly affected by the freshwater flow from the springs whose velocity dictates the distance from the sediment at which the Dead Sea water mixes in. The higher the flow, the further away from the sediment and the biofilms the mixing is, and *vice versa*. Thus, great fluctuations in the flow result in extreme and rapid fluctuations in the ambient biofilm salinity, as well as other physical–chemical parameters such as O_2_ concentration and pH (**Figure S1**).

In this study we used a metagenomics approach to hypothesize the osmoregulation strategy employed by the biofilm microorganisms living close to the groundwater outlets and to hypothesize whether or not such a strategy allows them to survive these salinity fluctuations. Despite being, on average, exposed to moderate salinities, we consider it unlikely that microorganisms constantly exposed to either freshwater or saturated brine in the freshwater springs of the Dead Sea can be osmotically balanced by exclusively using the salt-out strategy.

## Materials and methods

### Cultivation

The green biofilm samples were collected by scuba diving during 2011 (Ionescu et al., 2012). The cultures aimed at green sulfur bacteria isolation and enrichment were set using Dead Sea water diluted to 10% of the original salinity. The diluted brine was supplemented with 25 ml L^−1^ of PF-I solution (6.6 g/L of each KCl, MgCl_2_·6H_2_O, NH_4_Cl, and KH_2_PO_4_), 2.5 ml L^−1^ of PF-II trace element solution (Per L: 1 g of Na_2_-EDTA, 48 mg of CoSO_4_·7H_2_O, 400 mg of FeSO_4_·7H_2_O, 20 mg of ZnSO_4_·7H_2_O, 3 mg of CuSO_4_·5H_2_O, 4 mg of NiCl_2_·6H_2_O, 6 mg of Na_2_MoO_4_·2H_2_O, 6 mg of MnCl_2_·4H_2_O, and 60 mg of H_3_BO_3_), 2.5 ml L^−1^ of 1% NaHCO_3_, 2 mM sulfide (using Na_2_S·9H_2_O), 1 ml L^−1^ 10% Na-acetate, and 0.25 ml L^−1^ of 0.002% vitamin B_12_. No growth was observed when artificial Dead Sea water was used as a basis for media instead of the diluted lake water. The cultures were incubated at 30°C in dim light (10 µmol photons m^−2^ s^−1^).

### Nucleic acid extraction and sequencing

The DNA was extracted using a phenol–chloroform-based method, as described by Ionescu et al. (2012) using a basic (ca. pH 8) phenol solution. The DNA samples were sequenced on an Illumina HiSeq 2000 as 2 × 150 bp paired-end reads at MrDNA (Shallowater, TX, USA).

### Genome data analysis

All sequences were cleaned from adapter sequences and low-quality bases (Q < 15) using the Trimmomatic software (V 0.32) (Bolger et al., 2014). The DNA sequences were assembled using SPADES (V 3.14) (Bankevich et al., 2012), after which supervised binning was done using MetaBAT2 (Kang et al., 2019). The raw reads from the DNA sample were mapped using BBMap (V 38.0) to the contigs associated with each bin, after which the reads from each bin were assembled and binned individually. The process was repeated until the scaffold length was maximized and the contigs with obvious taxonomic mismatch were no longer present. The bin-refining module of Anvi’o (Eren et al., 2015) was used to refine some of the bins; however, having only one sample, the process led to minimal or no improvement. The scaffolds and the contigs of each bin were annotated separately using the Prokka pipeline (Seemann, 2014), the eggNOG database using eMapper (Huerta-Cepas et al., 2019), and RAST (Aziz et al., 2008; Brettin et al., 2015). The taxonomic annotation of the metagenome-assembled genomes (MAGs) was finally assigned using GTDB-Tk (V.2.3 DB version 207) (Chaumeil et al., 2022). The predicted proteins from each genome were further used for isoelectric point prediction using the IPC 2.0 software (Kozlowski, 2016).The genome-based phylogenetic trees of the obtained genomes and reference organisms were constructed using FastTree (Price et al., 2010) (v. 2.1.11; double precision; default parameters with gamma20 rescaling) from the multilocus alignment of the single-copy marker genes generated during the genome annotation using the GTDB-Tk tool (Chaumeil et al., 2022). In addition, the principal coordinate analysis (PCO) plots comparing SEED protein presence/absence between all the available genomes within the same genera to our retrieved MAGs were constructed. The pangenome comparisons were conducted using Anvi’o (Eren et al., 2015).

#### Data accessibility

The raw sequence data from the enrichment cultures and the MAGs were deposited into the National Center for Biotechnology Information (NCBI)’s Sequence Read Archive (SRA) (metagenomes) and GenBank^®^ databases under Bioproject PRJNA1033173 (https://www.ncbi.nlm.nih.gov/bioproject/PRJNA1033173). The MAG sequences are also available as part of the **Supplementary Material** of this paper to allow accessibility during NCBI internal processing.

## Results

### Characterization of the freshwater submarine springs

The freshwater springs in the Dead Sea were investigated and sampled during routine scuba dives between 2009 and 2014. White and green biofilms were observed throughout the sampling period (**Figure 1**) and various aspects of them have been studied and are described elsewhere (Ionescu et al., 2012; Häusler et al., 2014a; Häusler et al., 2014b; Häusler et al., 2014c).

These biofilms are exposed to great osmotic fluctuations, as changes in the groundwater inflows (**Figures S1A, S1AB**) are common and occur on a timescale of seconds to minutes, with extreme cases of complete ceasing of the freshwater flow being observed as well. Such fluctuations result in short- and long-term changes in salinity (**Figure S1C**) and oxygen concentration (**Figure S1C**) as well as in pH, temperature, and sulfide concentration. The underlying mechanism responsible for the inconsistent flow has not yet been elucidated and may vary between different springs, as the chemical analysis of the outflowing water shows different sources and different flow paths (Ionescu et al., 2012).

### Phylogenomic analysis

**Figure 2:**
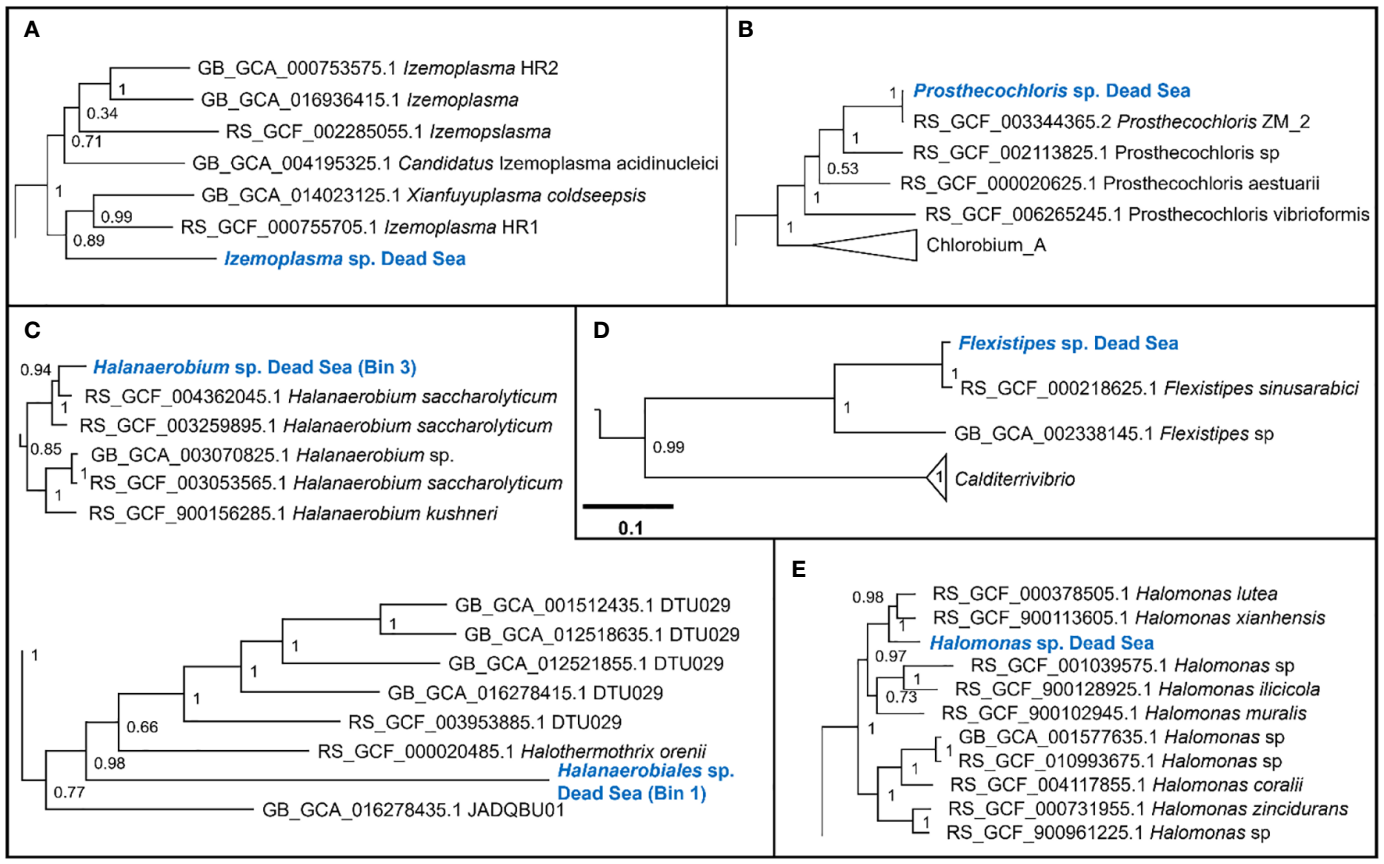
Sections of the whole-genome maximum likelihood phylogenetic trees showing the suggested taxonomic identity of the MAGs reconstructed from the Dead Sea cultures: **(A)** *Izemoplasma* sp.; **(B)** *Prosthecochloris* sp.; **(C)** *Halanaerobiales* spp.; **(D)** *Flexistipes* sp.; an **(E)** *Halomonas* sp. The trees were generated based on multisequence alignment, as generated by the GTDB-Tk tool using a bacterial marker set of 120 genes and 42 amino acids per marker (Chaumeil et al., 2022). The numbers next to the branches are the Shimodaira–Hasegawa support values (Shimodaira and Hasegawa, 1999; Guindon et al., 2010). The full trees are available in the **Supplementary Figures 2-6**.

**Figure 3:**
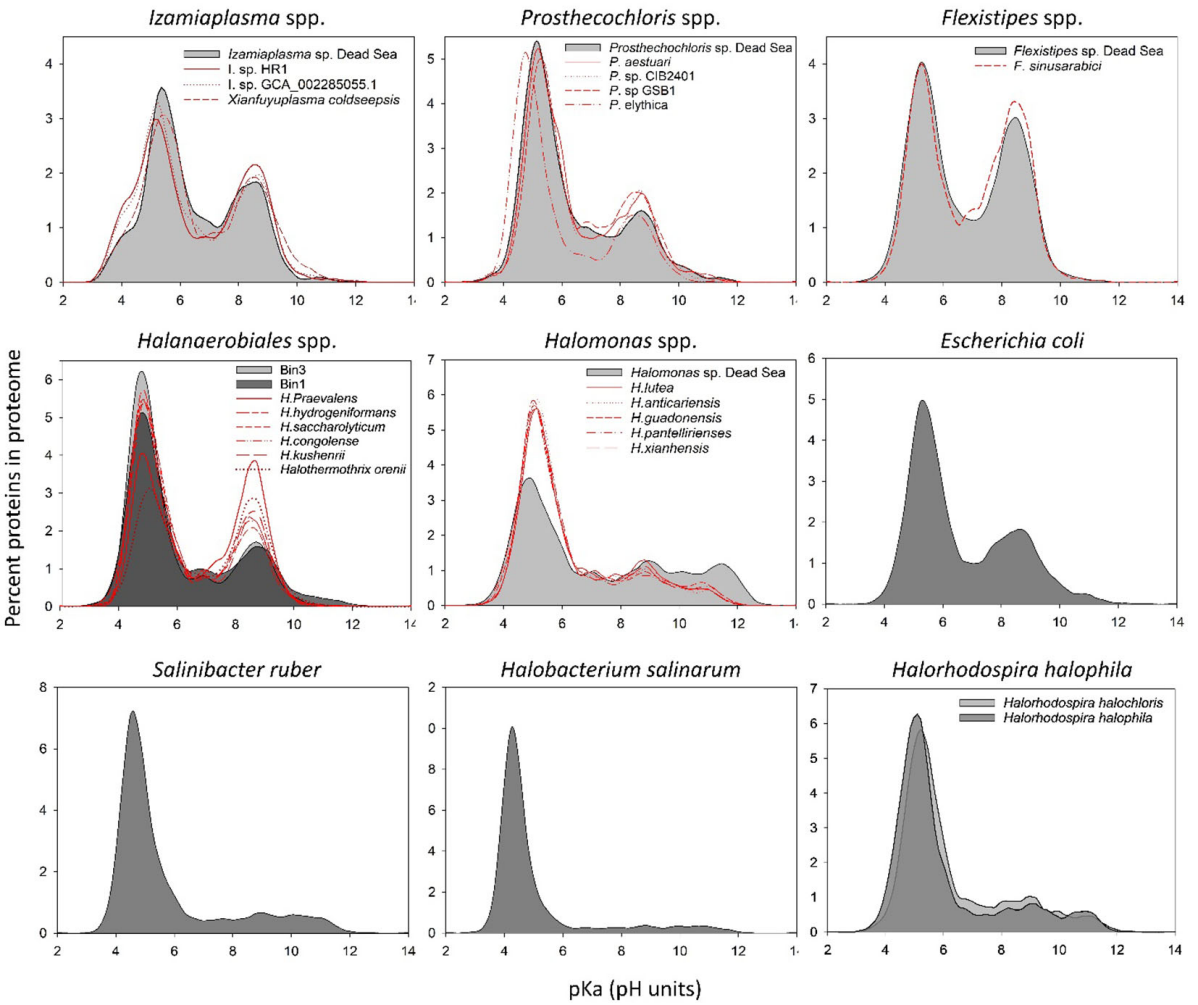
*In silico* whole-proteome isoelectric point profiles of the Dead Sea MAGs compared with several phylogenetically related organisms and model organisms.

We obtained six MAGs from the metagenomic library of the studied enrichment culture (**Figure 2**). These genomes were identified as *Izemoplasma* sp. (**Figure 2A**), *Prosthecochloris* sp. (**Figure 3B**), two different species of *Halanaerobiales* (**Figure 2C**), *Flexistipes* sp. (**Figure 2D**), and *Halomonas* sp. (**Figure 2E**). Assembly statistics and completeness are reported in **Table 1**.

**Table 1:** Assembly statistics.

Name	Comp.	Contam.	Assembly length	Number of contigs	N50	Max Contig
*Prosthecochloris* sp.	99.99	0.01	2.33 Mbp	47	84 Kbp	215 Kbp
*Flexistipes* sp.	99.88	0.05	2.47 Mbp	31	113 Kbp	381 Kbp
*Izemoplasma* sp.	99.01	0.00	1.68 Mbp	10	517 Kbp	571 Kbp
*Halanaerobium* sp. (Bin3)	63.22	2.40	1.89 Mbp	192	10 Kbp	31 Kbp
*Halanaerobiales* sp. (Bin1)	50.02	0.80	1.39 Mbp	157	9 Kbp	29 Kbp
*Halomonas* sp.	49.76	0.56	2.40 Mbp	417	5 Kbp	28 Kbp

Comp: Completness; Cont: Contamination.

The Dead Sea *Izmoplasma* genome clusters together with two genomes obtained from deep sea cold seeps: *Candidatus Izimaplasma* sp. HR2 from the Pacific Ocean hydrate ridge and *Xianfuyuplasma coldseepsis* from the South China Sea (**Figure 2A**).

The *Prosthecochloris* sp. from the Dead Sea falls into a cluster with sequences of other members of the same genus (**Figure 2B**). *Prosthecochloris* sp. strain ZM 2 (*Prosthecochloris ethylica* in GTDB) was isolated from the meromictic Green Cape Lake, *Prosthecochloris* sp. HL130 was isolated from the MgSO_4_-rich hypersaline lake Hot Lake, and *Prosthecochloris aestuarii* was isolated from the saline Lake Sasyk Sivash (Gorlenko et al., 1970). A comparison of 16S rRNA genes extracted from the metagenomic data (not shown) further shows similarity to *Prosthecochloris* sp. L21 and *Prosthecochloris* sp. LA53, which were obtained from environments with drastically different salinities. *Prosthecochloris* sp. L21 was obtained from Lake 21 on the atoll Kiritimati, which has a salinity of ca. 200 ppt (parts per thousand) (Spring et al., 2015). In contrast, *Prosthecochloris* sp. LA53 was obtained from the Hawaiian archipelago, where the salinity is ca. 30 ppt (Donachie et al., 2004).

The Dead Sea *Flexistipes* clusters together with a genome obtained from the Atlantis II deep brine pool in the Red Sea. The next-closest genome is fairly distant and was obtained from a large genome recovery project of the GTDB team, with no information on the environment from which it was recovered.

Within the *Halanaerobiales*, one Dead Sea strain (Bin1) clusters close to two genomes obtained from deep subsurface microbiomes found in extracted gas shales. The second genome (Bin3) has no known close relatives with sequenced genomes. The nearest know genome is that of *Halothermothrix orenii*, which was isolated from the hypersaline lake Chott El Guettar in Tunisia (Cayol et al., 1994).

The last genome that was obtained from the Dead Sea biofilm enrichment culture was of a *Halomonas* strain. This genome clustered together with two other *Halomonas* strains obtained from saline environments: *Halomonas lutea* (Wang et al., 2008) and *Halomonas xianhensis* (Zhao et al., 2012). Both *H. lutea* and *H. xuanhensis* were characterized by their ability to grow in an extremely broad salinity range (< 1%–20% salt).

### Analysis of isoelectric point profile of predicted proteomes from the metagenomic data

It has been suggested that changes at the level of protein amino acid composition and pIs (isoelectric points) represent a tool to predict the preferred habitat (i.e., salt adapted or salt free) of the different microorganisms (Cabello-Yeves and Rodriguez-Valera, 2019). To evaluate whether and which of the organism genomes that were isolated from the Dead Sea enrichment culture might have a high-salt-in-adapted proteome, we calculated the pIs of each predicted protein in the genomes (**Figure 3**). The Dead Sea *Prosthecochlois* sp., *Flexistipes* sp., and *Izemoplasma* sp. genomes were on par with data calculated from other available genomes. The pI profile generated from the *Prosthecochloris* genome is similar to that of non-halophilic organisms, such as *Escherichia coli*, and is slightly less acidic than its closest relative *P. ethylica*. In comparison, the Dead Sea *Flexistipes* sp. has more basic proteins than any of the other five genomes obtained. Both *Halanaerobium* sp. genomes have more acidic pI profiles than the related reference genomes. *Halomonas* reference genomes show that this genus is characterized by an overall acidic proteome, but it has more proteins with a high pI (i.e., > 10) than other organisms evaluated in this study. Nevertheless, the Dead Sea *Halomonas* genome appears less acidic than the references and contains more proteins with a pI above a pH of 10 (**Figure 3**). None of the bacteria enriched from the Dead Sea springs in this study has a comparable acidic proteome to those of *Halobacterium salinarum* and *Salinibacter ruber*, an archaeon and bacterium, respectively (**Figure 3**). *Halorhodospira halophila* and *H. halochloris* are two sister taxa that were reported to use the salt-in and salt-out strategies for osmoregulation, respectively (Deole et al., 2013; Oren, 2013a). Prior to genome sequencing, *H. halochloris* was thought not to have an acidic proteome, yet our analysis of the genome published by Singh et al. (Singh et al., 2014) shows otherwise (**Figure 3**).

### Genomic comparison

**Figure 4:**
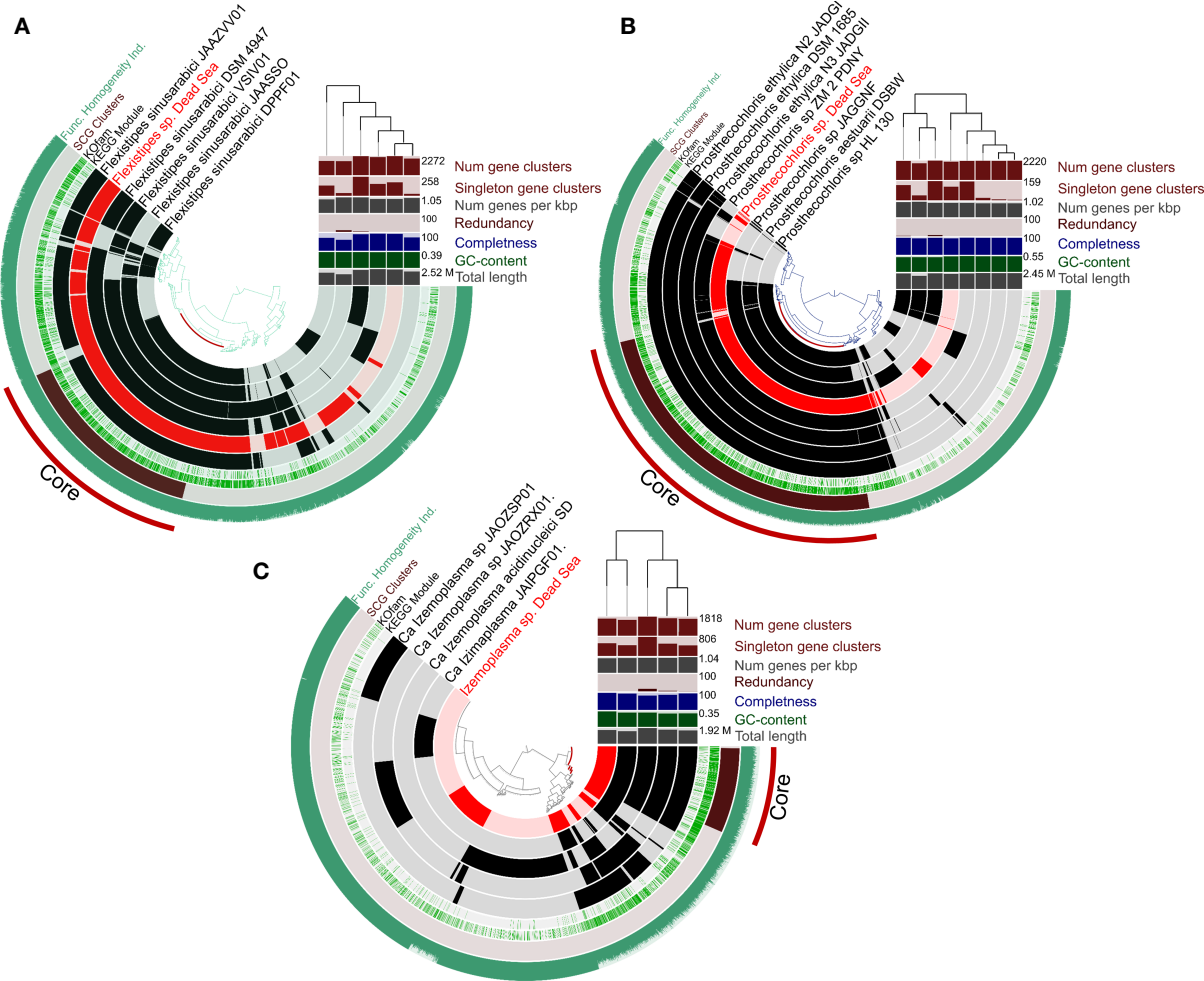
Circular representation of pangenomes of MAGs obtained from the Dead Sea springs enrichment culture and the most-closely available genomes in the database. Panels **(A-C)** show *Flexistipes* sp., *Prosthecochloris* sp., and *Isemoplasma* sp., respectively. The genomes of *Halanaerobiales* and *Halomonas* are not shown due to their relatively low level of completeness. The figures were generated using Anvi’o (Eren et al., 2015; Eren et al., 2020).

**Figure 5:**
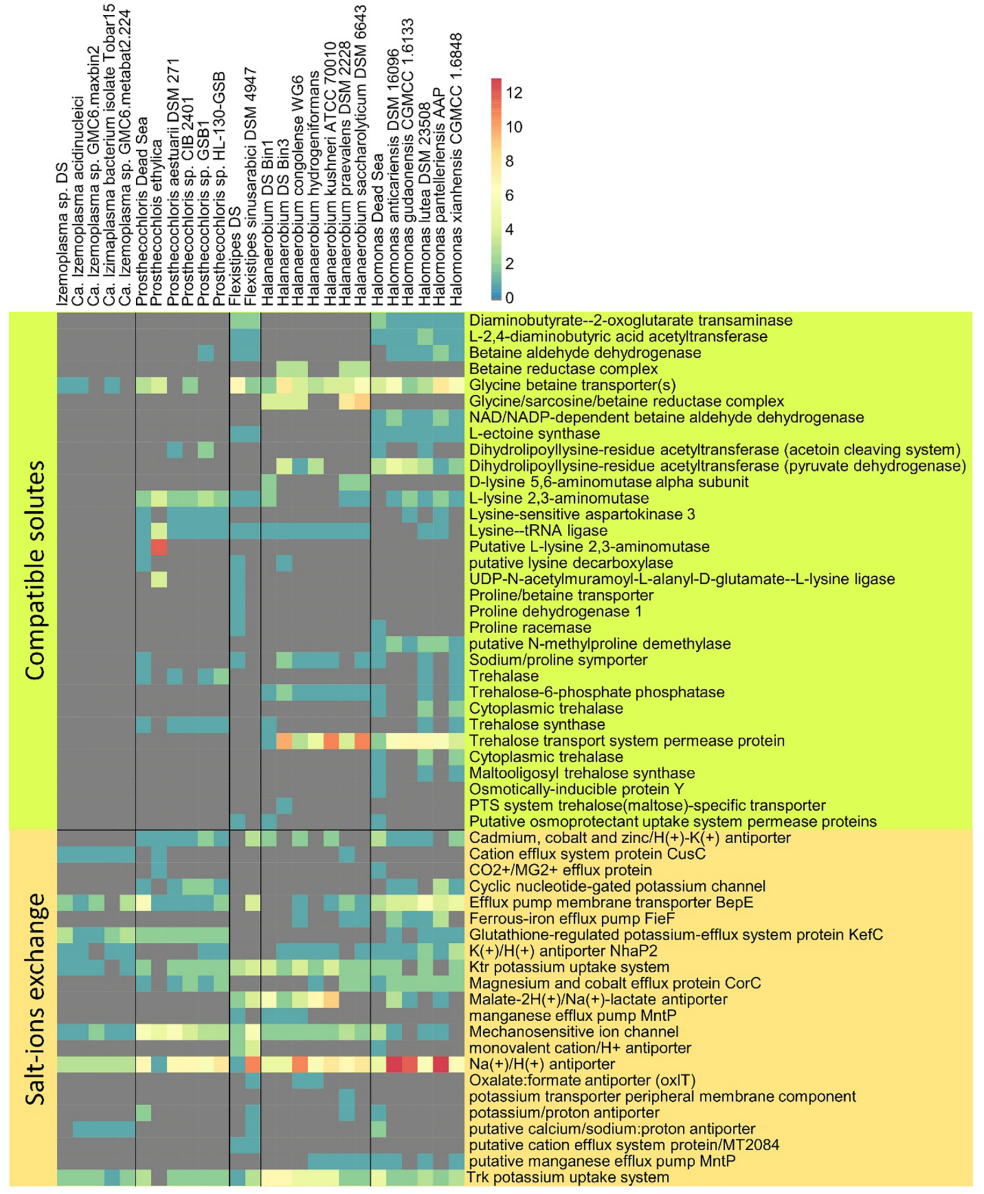
Osmotic response genes distribution across reference genomes and MAGs. The data are organized into compatible solute-related genes and genes involved in ion transport. The color scale indicates the number of genes identified per genome, related to a particular function (gray = 0).

An overall genome comparison of the Dead Sea MAGs and their closest relatives (**Figure 4**) reveals that based on the COGs (cluster of orthologous genes), the Dead Sea organisms share a large common core genome (See also **Table S2-S4**). A multi-environment SEED protein presence/absence PCO analysis for each taxon compared with their closest relatives to assess the uniqueness of Dead Sea MAGs compared with other habitats further confirms this (**Figure S1**). The smallest set of unique genes not shared with closely related genomes was observed in *Prostechochloris* sp. (141 gene clusters) (**Figure 4B**) and the largest in *Izamiaplasma* sp. (592 gene clusters) (**Figure 4A**), with *Flexistipes* harboring 216 unique gene clusters with 23, 28, and 63 unique COG functions for *Prosthecochloris* sp., *Flexistipes* sp., and *Izamiaplasma* sp., respectively. However, when inspecting functions there are fewer unique functions harbored by the Dead Sea organisms. It is important to state that, although the calculated completeness of the three MAGs presented in **Figure 5** is above 99%, the lack of some functions may still be a result of an incomplete genome. For *Prosthecochloris*, no unique pathway was observed, yet it appears to be entirely missing the F-type ATPase (**Table S1**). Compared with its nearest relatives, the Dead Sea *Izemoplasma* sp. harbors the pathway to produce riboflavin (vitamin B_2_), as well as the DAP (diaminopimelic acid) and the acetyl-DAP pathways for lysine synthesis (**Table S1**). The Dead Sea *Izemoplasma* sp. lacks several pathways when compared with some of its closest relatives; however, these are not exclusively present in all other genomes. When compared with the genomes of five different strains of *F. sinusarabici*, the Dead Sea *Flexistipes* sp. was found to lack the pathway for assimilatory sulfate reduction and biotin synthesis. In contrast, it was found to possess the commamox (complete oxidation of ammonia to nitrate) pathway, and almost exclusively possess the pathway for methionine degradation (**Table S1**).

In addition to comparing the presence and absence of KEGG (Kyoto Encyclopedia of Genes and Genomes) enzymes and modules, we compared the genomes for genes potentially involved in osmoregulation. Furthermore, while for a whole genome comparison we investigated only genomes with a high completeness, for the following analysis we investigated all six bins.

The Dead Sea *Izemoplasma* sp. genome, as well as other close relatives, were unique among the other obtained genomes in containing only glycine betaine transporters when considering the “salt-out” osmoregulation strategy (**Figure 5**). No significant dissimilarity between the Dead Sea genome and its closest relatives was observed with regard to osmoregulation genes.

Similarly to the *Izemoplasma* sp. genomes, the Dead Sea *Prosthecochloris* genome showed no remarkable differences to its closest relatives (**Figure 5**) with all species containing both genes for K^+^ uptake as well as synthesis and uptake of different compatible solutes. The genome comparison suggests *Prosthecochloris* sp. can utilize several types of compatible solutes but not Ectoine, while only the Dead Sea isolate appears to be capable of taking up proline via a sodium/Proline symporter.

Two different *Halanaerobium* genomes were obtained (**Figure 2**). Osmoregulation genes differ between the two genomes themselves as well as between the Dead Sea isolates and available genomes of other *Halanaerobium* sp. Both Dead Sea isolates contain more, and novel, cation transport genes as than the reference genomes, with the *Halanaerobium* Bin 3 genome being richer in cation transport genes than the *Halanaerobium* Bin 1. The *Halanaerobium* Bin 3 genome also contains a higher number of genes related to compatible solutes than *Halanaerobium* Bin 1 and most references. Among the compared *Halanaerobiales*, the Dead Sea *Halanaerobium* Bin 1 appears to be the only one able to synthesize trehalose rather than take it up. Nevertheless, missing genes may be a result of the relatively low completeness of these MAGs.

Comparing the Dead Sea *Flexistipes* genome to *F. sinusarabici* from a deep Red Sea brine, most of the differences are between genes related to compatible solutes (**Figure 4B**). Both *Flexistipes* sp. can synthesize ectoine, the most common compatible solute (Shivanand and Mugeraya, 2011), yet the Dead Sea isolate harbors additional genes for uptake of glycine betaine, proline, and other osmoprotectants. The complete absence of genes related to trehalose synthesis or metabolism suggests this genus does not utilize trehalose as a compatible solute.

The Dead Sea *Halomonas* isolate is enriched in cation transport genes as compared with the reference genomes (**Figure 4A**), and also harbors more diverse genes for compatible solute transport. In contrast, it has fewer copies of some of the genes for trehalose and glycine betaine transport. Like the other *Halomonas* it can synthesize ectoine but it can take up more osmoprotectants than most of the other *Halomonas*.

## Discussion

Halophilic organisms do not normally experience rapid, random, and drastic changes in salinity. However, the large biomass that is present in the Dead Sea underwater springs evidently shows that these organisms can cope with such atypical changes in salinity. In the case of Dead Sea underwater freshwater springs, it is evident by the large biomass present in the springs that these organisms cope with it. Additionally, previous studies have shown that these microbial biofilms are active. Sulfide oxidation was shown to take place in white biofilms (Häusler et al., 2014c) and oxygenic photosynthesis in brown and green biofilms found on sediments and rocks (Häusler et al., 2014b). In the latter case, a clear separation between diatoms and cyanobacterial biofilms was observed and was attributed to the organisms’ ability to withstand rapid versus long-term changes in salinity. Diatoms were shown to respond better to short-term, rapid fluctuations while the cyanobacteria are more tolerant to long exposures to undiluted Dead Sea water (Häusler et al., 2014b).

Using an enrichment culture targeting a green sulfur bacterium previously identified in the springs as *Prosthecochloris* sp. (Ionescu et al., 2012), we looked into the genomic potential for osmoregulation of several co-occurring bacteria to elucidate whether they adopt the salt-in or salt-out strategy.

We propose that the organisms inhabiting the Dead Sea springs do not exclusively use the salt-in strategy. The salt-in approach in which osmoregulation is handled via the uptake and release of K^+^ ions is energetically less expensive (Oren, 1999; Oren, 2011). Furthermore, the salt-in approach is expected to offer a more rapid response than the salt-out approach, as there is no need to synthesize or take up different organic compounds such as ectoine, glycine betaine, or trehalose. However, when compared with extreme halophiles, the proteome isoelectric point histograms of the Dead Sea MAGs did not resemble the extreme “salt-in”-using halophiles. Except for the two *Halanaerobiales* bins that appeared to harbor a more acidic proteome than their closest sequenced relatives, all bins had a similar profile to their phylogenetically close relatives. *Halorhodospira halophila* and *H. halochloris* are phylogenetically closely related or sister taxa. The former uses the salt-in strategy, whereas the latter uses the salt-out approach. Deole et al. (2013) have shown that *H. halophila* can withstand low-intracellular-K^+^ concentrations despite having an acidic proteome, thus showing that high-intracellular-salt concentrations are not essential for an acidic proteome to function. The acidic proteome of the sister taxa *H. halochloris*, which uses the salt-out strategy, further supports these conclusions and reveals that bacteria using the salt-out strategy may also have acidic proteomes. Nevertheless, to date, no salt-in utilizer has been identified with a non-acidic proteome. Therefore, it is unlikely that the Dead Sea spring organisms discussed here rely exclusively on this strategy.

The mechanistic of static versus dynamic osmoregulation in halophiles was seldom discussed, and when it was it particularly addressed Archaea (Becker et al., 2014). The last study suggested that halophilic Archaea utilizes both salt-in and salt-out strategies under fluctuating salinities, yet maintains an acidic proteome, as suited for their typical strategy. Our genome comparison analysis revealed that the isolates from the Dead Sea springs do not differ significantly from their closest relatives, and especially not with regard to known genes involved in osmoregulation. Overall, all the organisms appear to be able to make use of one (*Izemiplasma* sp.) or more compatible solutes, in parallel to the transport of ions, specifically K^+^. Interestingly, all genomes contain multiple mechanosensitive channels. These likely play a crucial role during sudden drops in salinity (Martinac, 2001; Blount and Iscla, 2020). In such events, the high intracellular concentration of salts and or solutes may lead to the rapid intake of water and eventually to cell burst. Thus, harboring multiple, and in some cases different, mechanosensitive channels can aid in the rapid release of solutes to prevent irreversible damage to the cells (Martinac, 2001; Bremer and Krämer, 2019; Blount and Iscla, 2020). During rapid increases in salinity, in parallel to the immediate uptake of K^+^ ions through one or more systems, all cells can start synthesizing solutes to increase the intracellular osmolarity while maintaining an overall low intracellular salt concentration (Bremer and Krämer, 2019).

The salinity optimum of the Dead Sea enrichment culture was determined based on the growth of the green sulfur bacterium *Prosthecochloris* at ca. 10% of Dead Sea water salinity. It may be that other members in the culture have a higher salinity optimum, as *Halanaerobium*, *Halomonas*, and *Flexistipes* are halophilic genera. Nevertheless, while both *Halanaerobium* genomes exhibit a more acidic proteome than other members of their genus, none of the bacteria studied here has a proteome as acidic as the salt-in users *Salinibacter ruber* or *Halobacterium salinarum*. The abundance of these organisms in the spring biofilms, alongside the monitored fluctuations in spring flow and salinity provide, strong evidence that these bacteria were coping well with the fluctuating environment.

Thus, we extend the conclusions presented by Becker et al. (2014) to include bacteria and suggest that organisms facing a dynamic osmo-environment make use of both the salt-in and salt-out strategies. At the same time, the proteome acidity of these organisms remains most compatible with the osmotic conditions most frequently encountered. This is further supported by the halophilic *Halorhodosphira halochloris*, which, despite losing the ability to accumulate high concentrations of potassium, maintains an acidic proteome typically suitable for the high-salinity, salt-in strategy.

The Dead Sea underwater spring biofilms provide a unique setting for the study of osmoregulation in bacteria, whereby these are subject to rapid and extreme changes in salinity in a natural way. Our study shows that in moderately halophilic, hyper-halotolerant bacteria, the “salt-in” and “salt-out” strategies are not mutually exclusive, as generally accepted. The response of microorganisms to extreme physical changes in such short periods in this ecosystem provides an opportunity for the real-time study of bacterial evolution on conditions likely similar to those during early life. Moreover, it is a source of information, physiological and genetic, for those looking to obtain strains that could adapt to such changing conditions in biotechnology.

## Data availability statement

The datasets presented in this study can be found in online repositories. The names of the repository/repositories and accession number(s) can be found in the article/**Supplementary Material**.

## Author contributions

DI: Conceptualization, Data curation, Formal Analysis, Investigation, Methodology, Project administration, Supervision, Validation, Visualization, Writing – original draft, Writing – review & editing. LZ: Data curation, Formal Analysis, Writing – original draft, Writing – review & editing. PC-Y: Data curation, Formal Analysis, Writing – review & editing. YT: Investigation, Resources, Writing – review & editing.
