# Supplementary material for: Extreme fluctuations in ambient salinity select for bacteria with a hybrid “salt-in”/”salt-out” osmoregulation strategy
Source: Front Microbiomes. 2024 Jan 8;2:1329925. doi: 10.3389/frmbi.2023.1329925 (PMC12993556; doi:10.3389/frmbi.2023.1329925)
Supplement: Supplementary file 1 [file DataSheet_1.zip › Ionescu_et_al_Dead_Sea_Supplementary_material-Final.docx]

**Extreme fluctuations in ambient salinity select for bacteria with hybrid “Salt-in” / ”Salt-out” osmoregulation strategy**

Ionescu Danny^1^*, Zoccarato Luca^2,3^, Pedro J. Cabello-Yeves^4,5,6^, Tikochinski Yaron^8^

**Supplementary material**


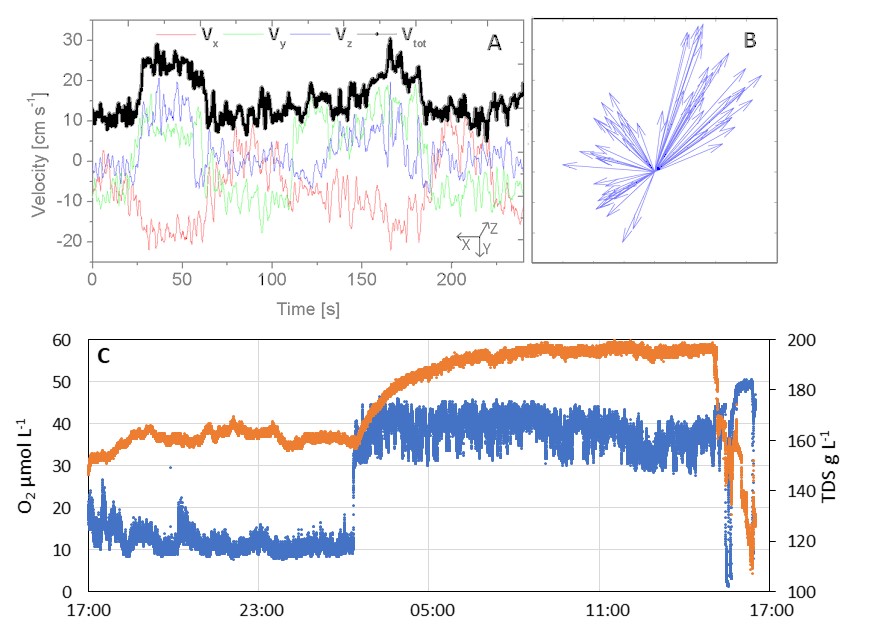


**Figure S1.** Example of flow velocities measured in situ in the center of a spring at a vertical distance of 2 cm. Panel (A) shows all three cartesian components of the velocity vector (z-component corresponds to the vertical direction), as well as the vector magnitude, as a function of time. Panel (B) shows the projections of the velocity vector in the vertical plane (the time axis is collapsed). Panel (C) shows O_2_ (blue) and salinity (orange) measurements over 24 h. Continuous fluctuations are clearly visible on the O_2_ profile. The slower response time of the salinity sensor masks the short-term fluctuations. Measurements were taken 1 s apart. The rapid increase in oxygen concentration and salinity at ca. 2 AM, reflect a sudden decrease in spring water flow.


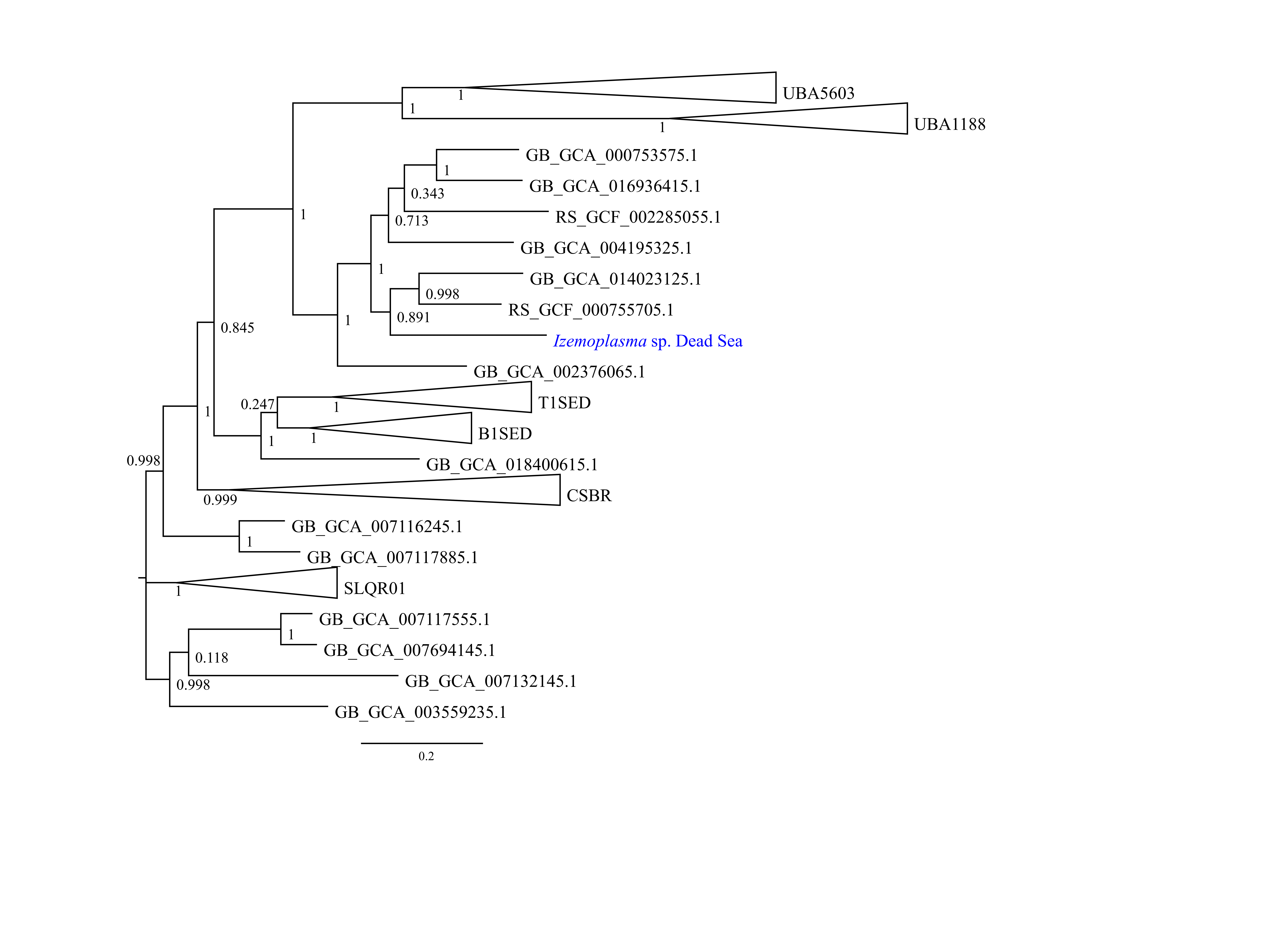


**Figure S2.** Whole-genome maximum likelihood phylogenetic tree showing the suggested taxonomic identity of the *Izemoplasma* sp metagenome assembled genomes reconstructed from the Dead Sea culture. The tree was generated based on multisequence alignment as generated by the GTDB-TK tool using a bacterial marker set of 120 genes and 42 amino acids per marker (Chaumeil et al., 2022). The numbers next to the branches are the Shimodaira-Hasegawa support values (Shimodaira and Hasegawa, 1999; Guindon et al., 2010).


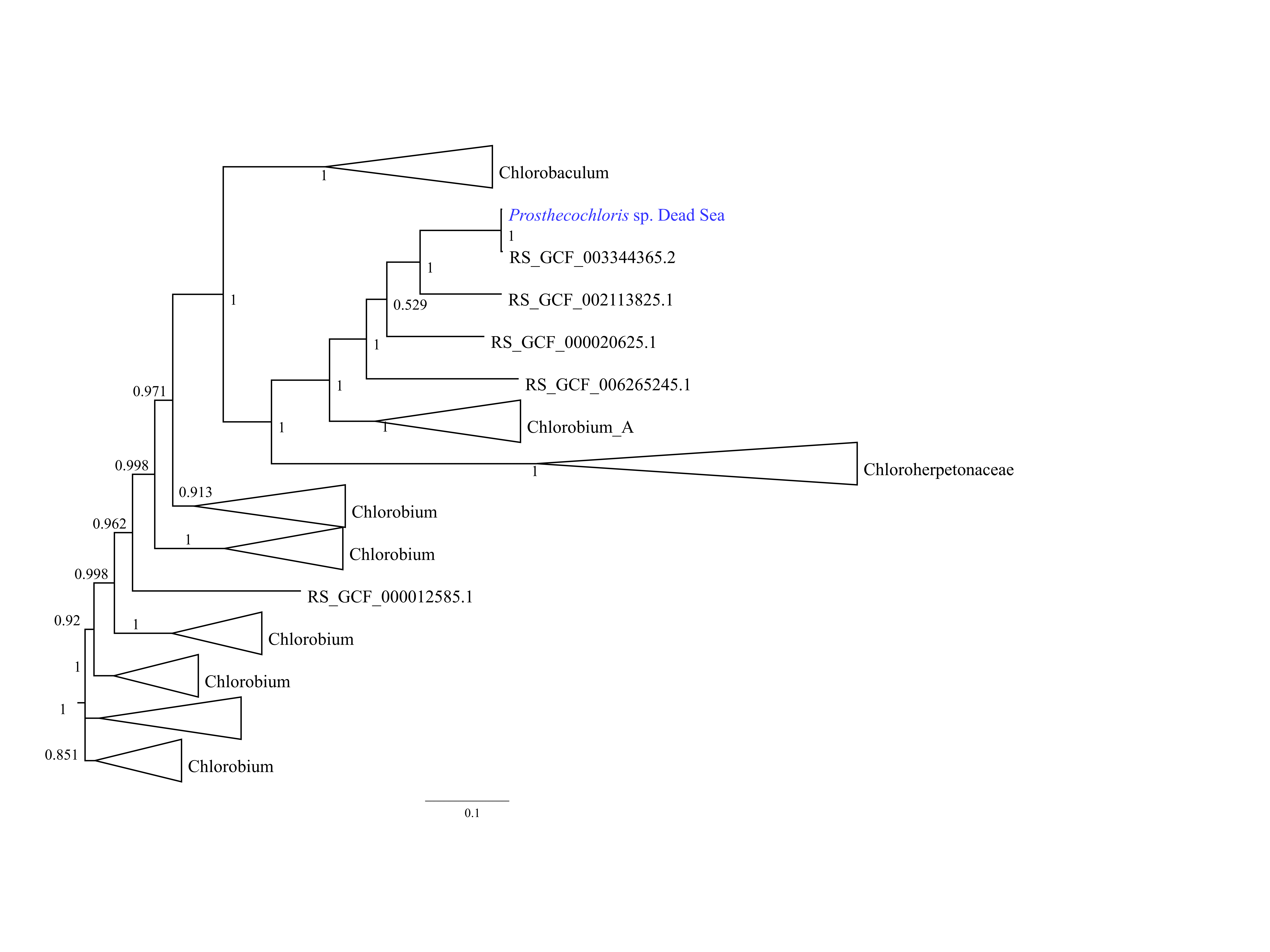


**Figure S3.** Whole-genome maximum likelihood phylogenetic tree showing the suggested taxonomic identity of the *Prosthecochloris* sp metagenome assembled genomes reconstructed from the Dead Sea culture. The tree was generated based on multisequence alignment as generated by the GTDB-TK tool using a bacterial marker set of 120 genes and 42 amino acids per marker (Chaumeil et al., 2022). The numbers next to the branches are the Shimodaira-Hasegawa support values (Shimodaira and Hasegawa, 1999; Guindon et al., 2010).


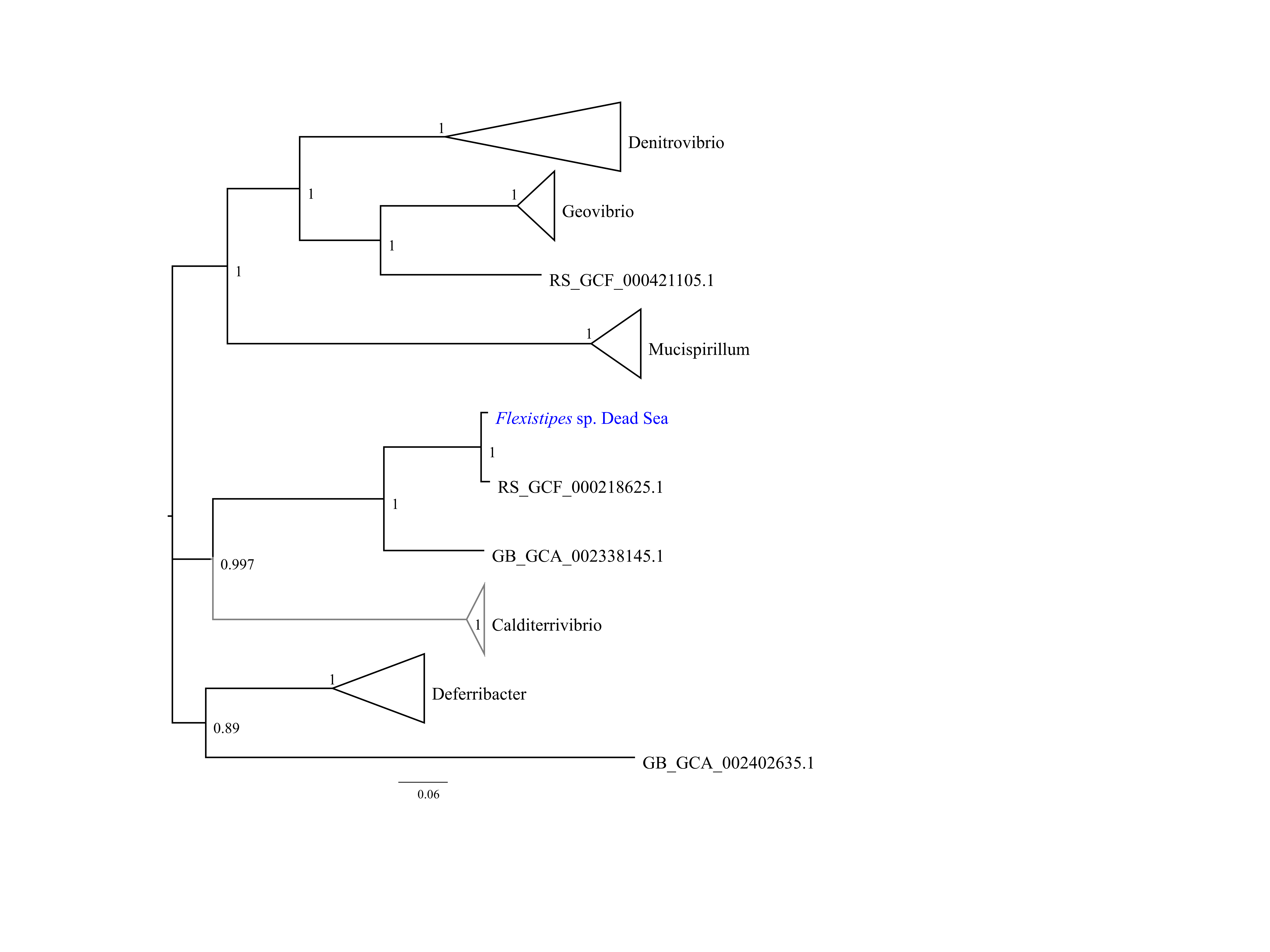


**Figure S4.** Whole-genome maximum likelihood phylogenetic tree showing the suggested taxonomic identity of the *Flexistipes* sp metagenome assembled genomes reconstructed from the Dead Sea culture. The tree was generated based on multisequence alignment as generated by the GTDB-TK tool using a bacterial marker set of 120 genes and 42 amino acids per marker (Chaumeil et al., 2022). The numbers next to the branches are the Shimodaira-Hasegawa support values (Shimodaira and Hasegawa, 1999; Guindon et al., 2010).


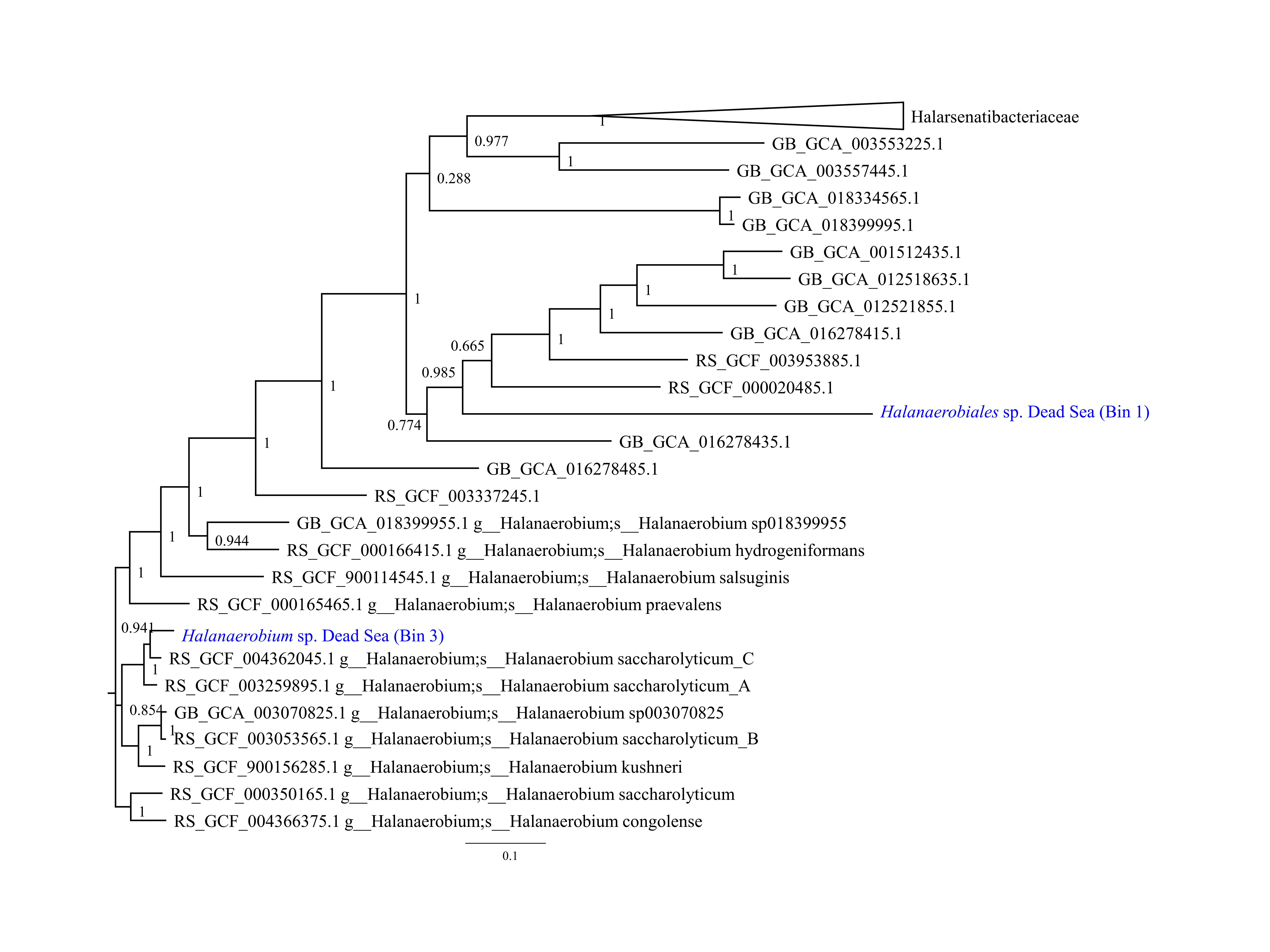


**Figure S5.** Whole-genome maximum likelihood phylogenetic tree showing the suggested taxonomic identity of the *Halanerobiales* spp metagenome assembled genomes reconstructed from the Dead Sea culture. The tree was generated based on multisequence alignment as generated by the GTDB-TK tool using a bacterial marker set of 120 genes and 42 amino acids per marker (Chaumeil et al., 2022). The numbers next to the branches are the Shimodaira-Hasegawa support values (Shimodaira and Hasegawa, 1999; Guindon et al., 2010).


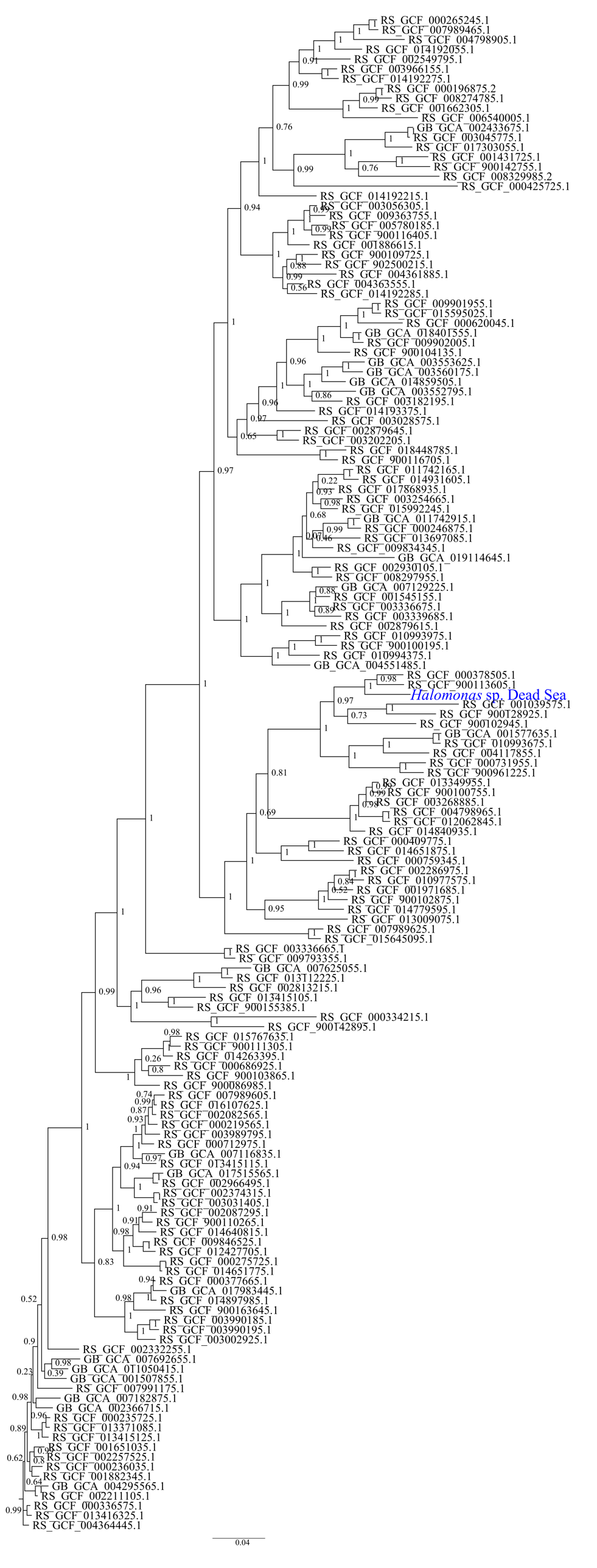

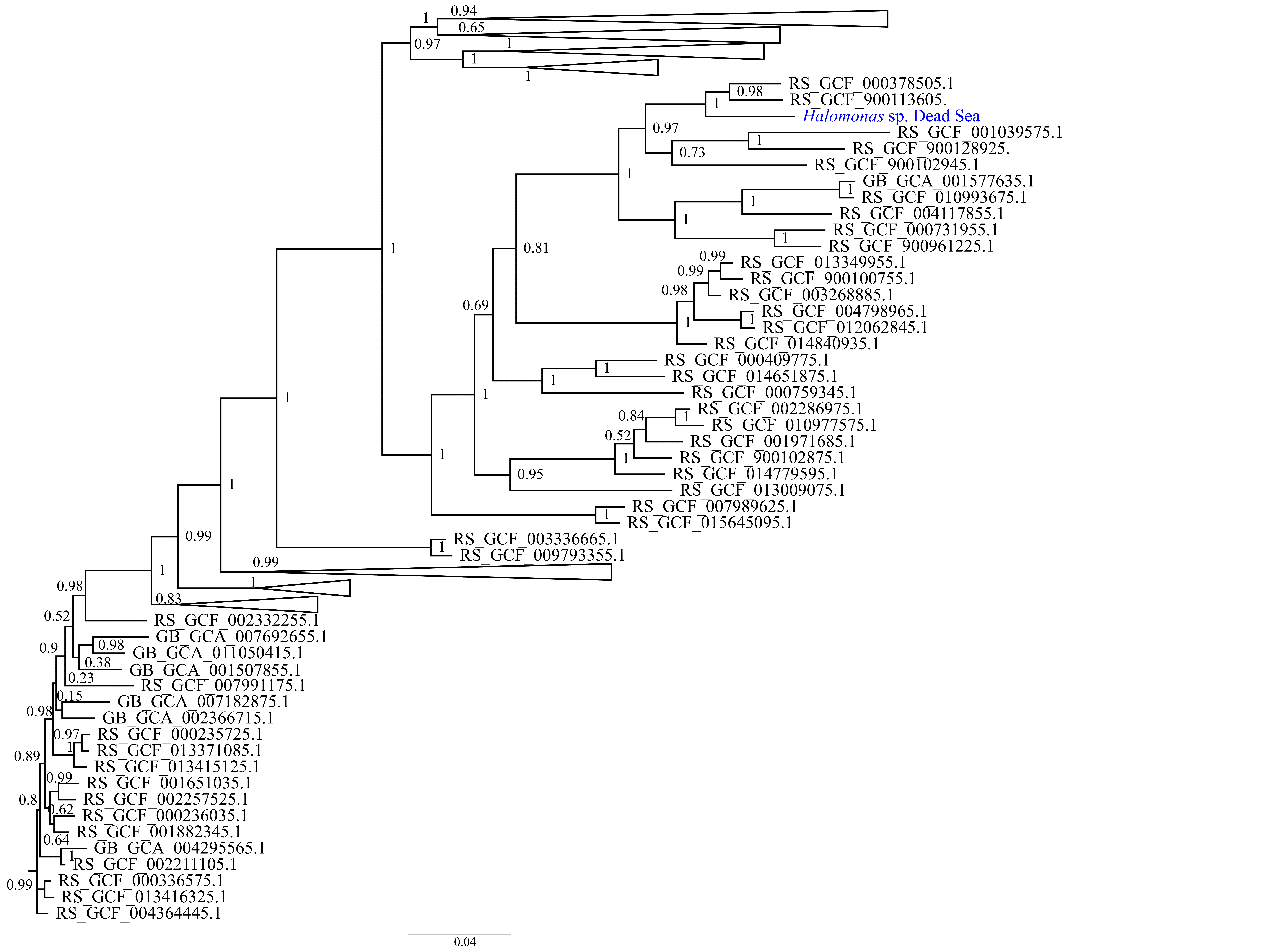


**Figure S6.** Whole-genome maximum likelihood phylogenetic tree showing the suggested taxonomic identity of the *Halomonas* sp. metagenome assembled genomes reconstructed from the Dead Sea culture. The tree was generated based on multisequence alignment as generated by the GTDB-TK tool using a bacterial marker set of 120 genes and 42 amino acids per marker (Chaumeil et al., 2022). The numbers next to the branches are the Shimodaira-Hasegawa support values (Shimodaira and Hasegawa, 1999; Guindon et al., 2010). Given the large number of available *Halomonas* spp. genomes, the insert provides a focused view on the Dead Sea MAG.


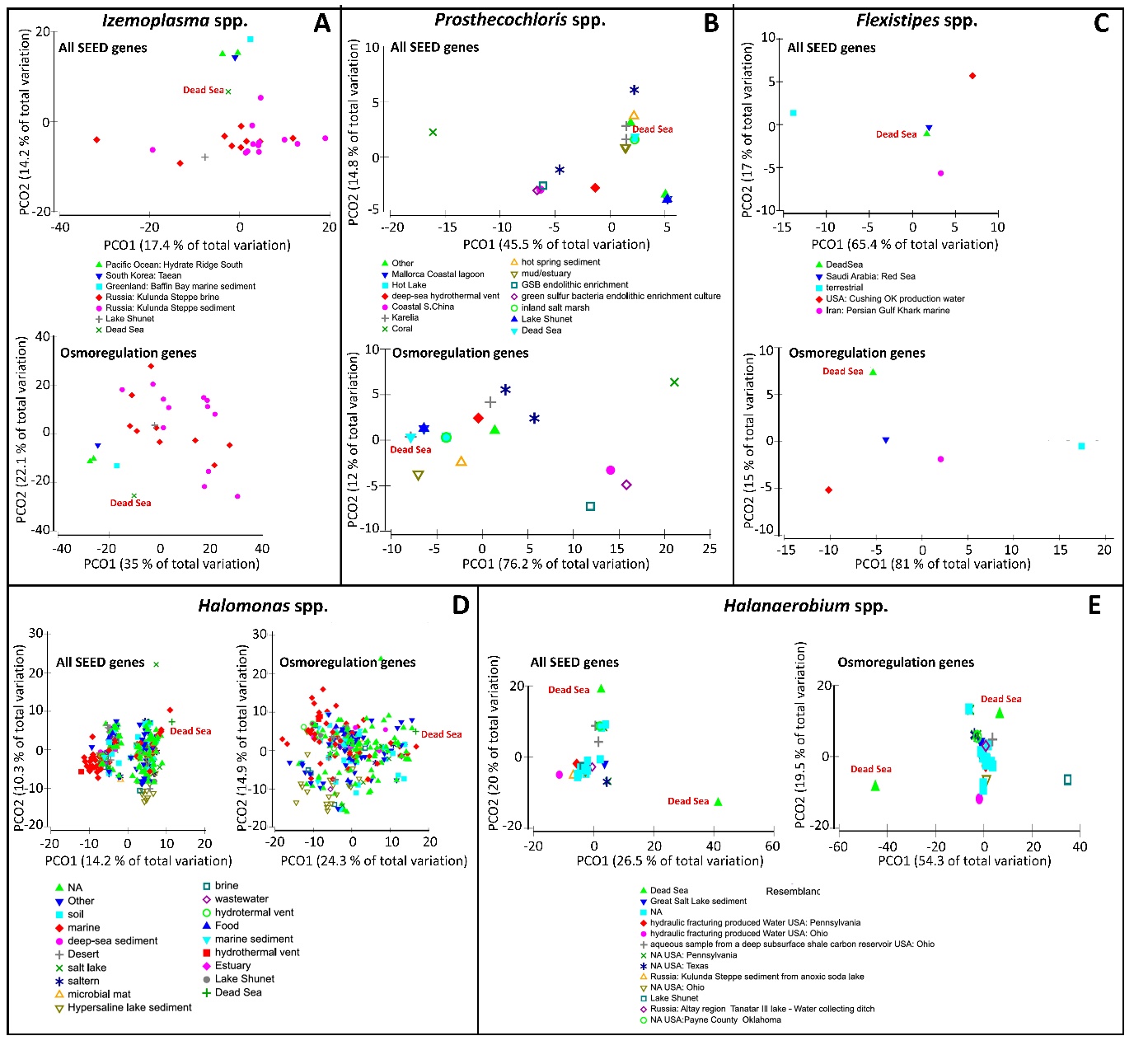


**Figure S7**. Principle component analysis of present or absent genes in the Dead Sea spring originating genomes compared to their other genomes in their genus. Analyses were done for all genes classified with SEED classification (Overbeek et al., 2014) and separately for a subset of those genes related to osmoregulation. For this annotation, the sequences for comparison and the SEED annotation of the Dead Sea bacteria were obtained from the Bacterial and Viral Bioinformatics Reference Center (https://www.bv-brc.org/).

**Table S1.**

Comparison of KEGG orthologs and KEGG Modules of the three MAGs with the highest genome completeness. *Izemoplasma* sp., *Prosthecochloris* sp., *Flexistipes* sp.

**Table S2.**

ANVI’O (Eren et al., 2015) generated gene cluster comparison of *Flexistipes* spp. COG and KEGG annotation is provided where genes could be annotated.

**Table S3.**

ANVI’O (Eren et al., 2015) generated gene cluster comparison of *Prosthecochloris* spp. COG and KEGG annotation is provided where genes could be annotated.

**Table S4.**

ANVI’O (Eren et al., 2015) generated gene cluster comparison of *Izemoplasma* spp. COG and KEGG annotation is provided where genes could be annotated.

**Dead_Sea_MAGs.zip**

File containing the contig sequences of the 6 MAGs obtained from the Dead Sea springs enrichment culture.
